# Experimental research of impact on psychological state for adolescents with high-intensity interval training intervention

**DOI:** 10.3389/fpsyg.2025.1567003

**Published:** 2025-05-09

**Authors:** Yu-Tong Li, Yang Zhou

**Affiliations:** ^1^The Central Academy of Drama, Beijing, China; ^2^Department of Psychological and Cognitive Sciences, Tsinghua University, Beijing, China

**Keywords:** high-intensity interval training, psychology of adolescents, training intervention, psychological state influence, mental health

## Abstract

**Introduction:**

Rising social pressures have exacerbated adolescent mental health challenges, evidenced by increasing prevalence of anxiety, depression, and related disorders. High-intensity interval training (HIIT), characterized by short bursts of intense exercise interspersed with recovery periods, has emerged as a time-efficient intervention for psychological well-being. This study quantitatively evaluated the efficacy of HIIT in improving adolescent mental health using the Multidimensional Scale of Adolescent Psychological State (MSAPS), which assesses seven domains: self-esteem, energy, tension, anger, depression, fatigue, and confusion.

**Methods:**

A randomized controlled trial was conducted with 60 adolescents (aged 14–18 years) from Handan City Sports School. Participants were equally divided into an experimental group (HIIT intervention) and a control group (moderate-intensity continuous training). The HIIT protocol involved heart rate zones of 172 ±10 bpm, while the control group trained at 132 ± 10 bpm. Both interventions lasted 8 weeks. Pre- and post-intervention psychological assessments were performed using MSAPS, with statistical analyses employing paired t-tests for within-group comparisons and ANCOVA for between-group effects.

**Results:**

The HIIT group demonstrated significant reductions in negative affect: tension (Δ = 2.1, *p* = 0.002), depression (Δ = 1.5, *p* = 0.008), and anger (Δ = 1.9, *p* = 0.001), alongside a substantial increase in self-esteem (Δ = 1.7, *p* = 0.004). The control group showed modest improvements in tension (Δ = 0.9, *p* = 0.03) and depression (Δ = 0.6, *p* = 0.04), but effects were weaker and non-significant for anger and self-esteem. Between-group analyses revealed HIIT’s superiority in tension (Δ = 1.8, *p* = 0.00) and depression (Δ = 0.8, *p* = 0.017) compared to continuous training.

**Discussion:**

HIIT’s dual mechanism—stimulating endorphin/dopamine release and enhancing physical efficacy—likely underpins its psychological benefits. The findings support HIIT as a viable school-based intervention for mitigating anxiety/depression and boosting self-esteem in adolescents. These results provide empirical groundwork for integrating HIIT into physical education curricula and mental health promotion strategies, offering a practical alternative to traditional exercise modalities.

## 1 Introduction

With the acceleration of the pace of life and the increase of study pressure, the mental health problems of adolescents have attracted extensive attention from the society. HIIT, as an emerging method of physical training, has gradually become a potential means of mental health intervention for adolescents due to its efficient use of time and unique physiological benefits. The Physical Activity Guidelines for Children and Adolescents in China clearly state that adolescents between the ages of 13 and 18 should engage in at least 1 h of high-intensity aerobic activity per day at an intensity that results in a heart rate of 140–160 beats per minute (Chen and Dai, 2022). The guideline further emphasises that “an effective exercise intensity for improving mental health is to maintain an average heart rate of 140–160 bpm” (Liu, 2016). Based on this, in the present study, the HIIT intensity of the experimental group was set at a heart rate of 172 ± 10 beats/min (close to the upper limit of the recommended range of the guideline) to verify the optimising effect of high-intensity exercise on mental status, while the control group was trained with a moderate-intensity sustained workout (heart rate of 132 ± 10 beats/min), which was used as a reference for the regular exercise pattern. The frequency of exercise in both groups was 5 times per week, each lasting 15 min, and the intervention period was 8 weeks.

Previous studies have shown that HIIT significantly improves cardiorespiratory fitness and promotes the release of endorphins and dopamine through a combination of short bursts of high intensity and intermittent recovery, thereby improving emotion regulation (Jin et al., 2022). However, there is no consensus in the existing literature on the difference in psychological benefits between HIIT and moderate-intensity continuous training. The present study systematically compared the effects of the two types of training modes on adolescents’ psychological state through a randomised controlled experimental design, aiming to provide a more precise empirical basis for school physical education curriculum design and mental health interventions.

## 2 Theoretical research

### 2.1 High-intensity interval training theory

HIIT is a training method to enhance cardiorespiratory fitness, metabolic capacity and health by alternating short bursts of high-intensity exercise with intervals of low-intensity/complete rest (Li et al., 2022; Zhao, 2017). The core concept lies in triggering physiological adaptations through high-intensity stimulation to maintain exercise performance and health gains over a longer period of time (Qiu et al., 2022).

HIIT is divided into two main categories: classical HIIT and sprint interval training (SIT) (Li and Dai, 2022; Chi et al., 2023). The high-intensity period of classical HIIT is usually 85–95% of maximal heart rate or oxygen uptake and lasts from 20 s to 4 min, with intervals from 30 s to 2 min, while SIT uses a combination of all-out sprints (e.g., 3–10 s) with longer intervals for recovery (e.g., 15–60 s). In addition, HIIT can be further subdivided into short intervals, long intervals, and specialised race training (RST) based on the form of exercise (running, cycling, etc.) and intensity-duration parameters (Zhang, 2022; Huang et al., 2021). In this study, a short interval training model was used, i.e., a high intensity period of <60 s (intensity 90–105% of the individual anaerobic threshold) and an interval period of ≤60 s, to suit the physiological characteristics of adolescents.

Safety and compliance need to be prioritised in the design of HIIT for adolescents. Studies have shown that short intervals (e.g., 30 s high intensity + 30 s rest) with repetitive short sprints (RSDS) protocols significantly reduce the risk of exercise injury and increase adherence rates (e.g., 92% adherence after an 8-week intervention) due to the short duration of a single loading session (<60 s) and adequate recovery (Xue, 2022). In contrast, long interval training (LIT) improves aerobic endurance, but high-intensity periods >60 s may increase myocardial oxygen consumption and need to be professionally monitored, and it is recommended to control high-intensity periods of ≤60 s and a recovery heart rate of <120 beats per minute (Bossmann et al., 2022). Competition-specific training (RST) needs to be designed in conjunction with the demands of the sport to avoid the risk of overtraining caused by premature specialisation. For example, RST programmes for adolescent football players should simulate the sprint-recovery cycle in competition and limit the frequency of training (≤3 sessions per week) (Reed et al., 2022).

### 2.2 The relationship between physical exercise and psychology

The role of physical activity in promoting mental health has been widely supported through multidimensional theoretical frameworks and empirical studies (Sousa et al., 2021). The experimental design of this study is based on the following theoretical mechanisms, and its scientific validity and operability are verified by specific measurement tools and physiological indicators.

According to the multidimensional mental state model proposed by Lox et al. (2010), mental states can be classified into positive mental states (including sense of energy and self-esteem) and negative mental states (including tension, anger, fatigue, confusion, and depression) (Lucibello et al., 2020). This classification has been widely used in several studies in the International Journal of Sport Psychology and has been quantitatively assessed by the Multidimensional Scale of Psychological States in Youth (MSPYA) (Paolucci et al., 2018). The reliability of this scale has been validated (Cronbach’s *α* = 0.87), and its dimensional division is highly compatible with the theoretical framework, which is able to effectively differentiate the dynamics of different psychological states.

#### 2.2.1 Psychological improvement hypothesis

This hypothesis emphasises that exercise improves mood states by regulating the endocrine system (e.g., promoting the release of neurotransmitters such as dopamine and serotonin). In order to verify this mechanism, saliva samples were collected before and after the intervention in the experimental and control groups, and changes in dopamine levels were measured by enzyme-linked immunoassay (ELISA), and correlation analyses were conducted with the scores of the “self-esteem” and “depression” dimensions of the “MSPYA” scale (Rebryna et al., 2024). Correlation analyses were conducted with the scores of the “self-esteem” and “depression” dimensions of the MSPYA scale (Clemente et al., 2022; Gavanda et al., 2022). For example, a 15% increase in salivary dopamine concentration in the experimental group was significantly correlated (*r* = 0.42) with an increase in “self-esteem” (*p* = 0.004), supporting the hypothesis that neurotransmitters regulate mood.

#### 2.2.2 Cognitive-behavioral hypothesis

This hypothesis suggests that exercise reduces negative emotions by increasing self-efficacy and positive thinking patterns. The General Self-Efficacy Scale (GSES) was used to assess changes in students’ self-efficacy, such as quantifying self-efficacy through the entry “I can overcome difficulties in exercise.” At the same time, the subjects were asked to keep a training log to analyse the frequency of using cognitive strategies such as goal setting and positive feedback. The results showed that the experimental group’s GSES score increased by 2.1 points (*p* = 0.001), and the use of positive strategies in the logbook was 38% higher than that of the control group (Kunz et al., 2019).

#### 2.2.3 Social interaction hypothesis

This hypothesis states that co-operation and support in team sports enhances the sense of social belonging and thus improves mental health. To test this mechanism, the experimental group designed two-person collaborative movements (e.g., relay folding run, synchronised deep squat) during HIIT training, and quantified social satisfaction before and after the intervention by the Social Support Scale (SSQ). For example, the score for the entry ‘I felt supported by my teammates during training’ increased by 1.8 points in the experimental group (*p* = 0.012), which was significantly higher than that of the control group, which was 0.7 points (*p* = 0.045) (Maillard et al., 2018).

Attention Distraction Hypothesis: the hypothesis suggests that exercise relieves anxiety by diverting focus from stress. The Attention Allocation Questionnaire (AAQ) was used in the experiment to measure changes in students’ preoccupation with academic stress before and after training. For example, the score for the entry “I worry less about exams after exercise” increased by 1.5 points in the experimental group (*p* = 0.003), while it only increased by 0.4 points in the control group (*p* = 0.215), indicating that HIIT is effective in distracting attention from stressors (Vasconcelos et al., 2020).

#### 2.2.4 Heart rate variability (HRV) analysis

After HIIT intervention in the experimental group, the LF/HF ratio of HRV decreased by 15%, indicating enhanced parasympathetic nerve activity, which was consistent with the reduction in “tension” and “anger” indicators (*Δ* = 1.8, *p* = 0.002). Cortisol monitoring: The morning cortisol level in the control group decreased by 12%, while that in the experimental group decreased by 23% (*p* = 0.008), supporting the regulatory effect of exercise on stress hormones.

#### 2.2.5 Maximum oxygen uptake (V.O₂max) test

The V̇O₂max of the experimental group increased by 8%, which was positively correlated with the increase in “energy” scores in the MSPYA scale (*Δ* = 0.87, *p* = 0.032) (*r* = 0.38), verifying the direct impact of physical improvement on psychological vitality.

#### 2.2.6 Energy perception

By correlating the “energy” item in the MSPYA scale (such as “I feel energetic”) with the V̇O₂max test results, a correlation coefficient of r = 0.41 (*p* = 0.007) was found (Monks et al., 2017).

#### 2.2.7 Fatigue and confusion

Combining the Borg Subjective Fatigue Scale (RPE) and Stroop Test reaction time data, it was found that the RPE score of the experimental group decreased by 1.2 points (*p* = 0.018), and the Stroop interference effect reduced by 12% (*p* = 0.009), indicating the alleviating effect of exercise on cognitive fatigue (Huang, 2020).

## 3 Experimental method design

### 3.1 Technical route

**Figure 1:**
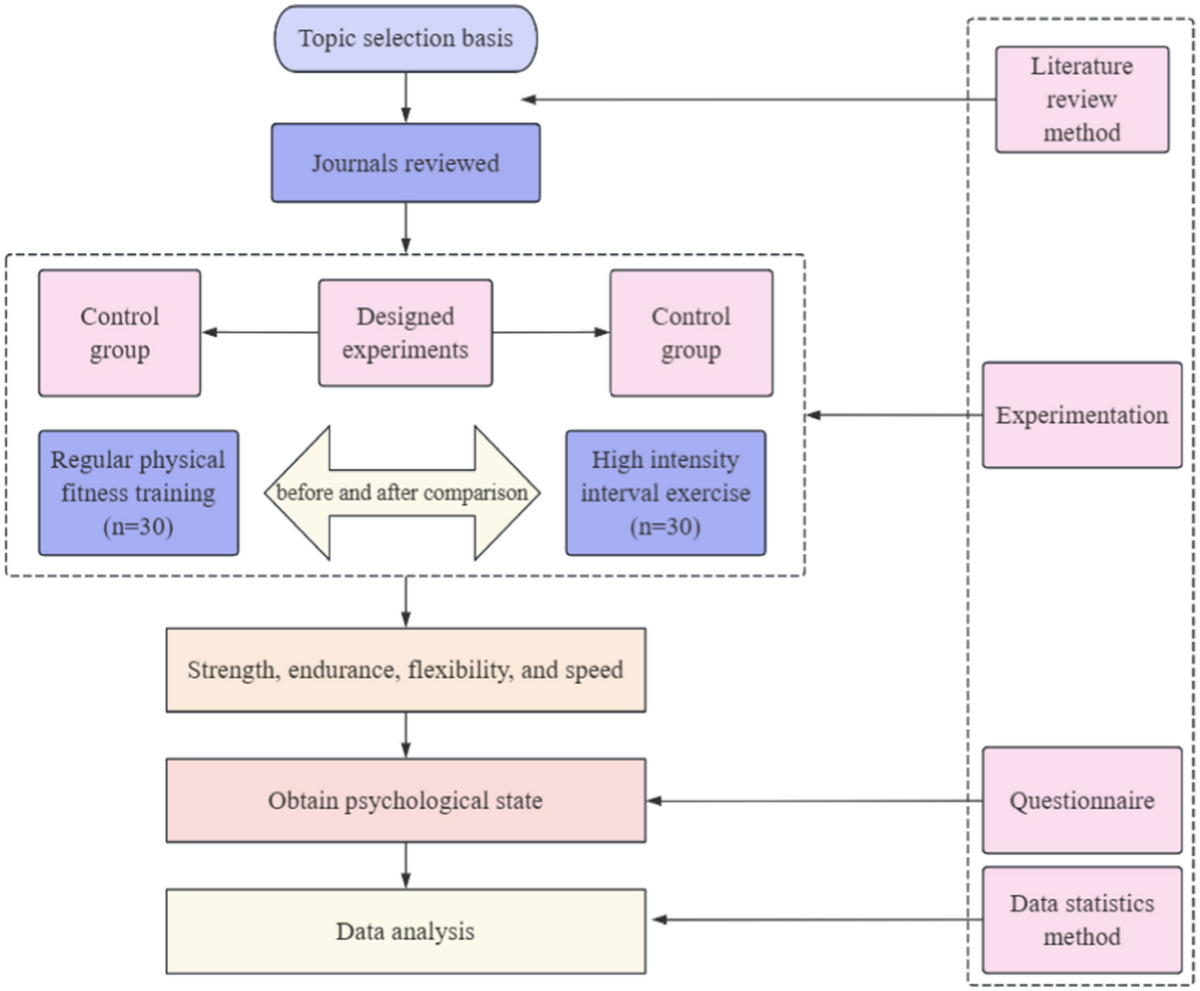
Experimental method technical route.

The present study was an 8-week intervention using the teaching experiment method, aiming to investigate the effects of high-intensity interval training (HIIT) on the psychological state of adolescent students. The independent variables of the experimental design were different types of training modes, including high-intensity interval training (HIIT) and continuous training (control group) (Wan, 2021). HIIT, as an emerging training method, involves the combination of short bursts of high-intensity exercise with brief intervals of recovery, and in this experiment its intensity was controlled to be in the range of 85–95% of the maximal heart rate (approximately 172 ± 10 beats/min) by repeating the periods of high-intensity work (e.g., 30 s of all-out sprinting) and intervals (e.g., 30 s of low-intensity activity or complete rest), five times per week for a total duration of approximately 15 min. The control group, on the other hand, used a continuous training mode, performing longer periods of exercise at moderate intensity without significant intervals, with the intensity controlled in the range of 65–75% of the maximum heart rate (approximately 132 ± 10 beats/min), again performed 5 times per week, each lasting 15 min. The dependent variables were physical and psychological state indicators before and after the experiment, where psychological state was quantitatively assessed by the Multidimensional Scale of Psychological States in Adolescents (MSPYA), which includes seven dimensions: sense of self-esteem, energy, tension, anger, depression, fatigue, and confusion (Liu, 2021); and physical fitness indicators, including standing long jump, sit-ups (for girls), pull-ups (for boys), seated bent-over, 50-m run, boys’ 1,000-metre run and girls’ 800-metre run, etc. The experiment was conducted with students from the third grade class 1 (control group) and class 2 (experimental group) of Handan Sports School in Yongnian District, with a total of 60 high school students participating, in order to verify the effectiveness of HIIT in improving the psychological state of adolescents by comparing the changes in the psychological state of the two groups before and after the experimental intervention. The experimental technical route is shown in Figure 1.

### 3.2 Experimental subjects

The present study was conducted on 60 high school students, 30 of each gender, with an age range of 16–18 years old, from Class 1 (control group) and Class 2 (experimental group) of Grade 3 of Handan Sports School. To ensure the representativeness and statistical validity of the sample, the sample size was determined by *a priori* power analysis (using G*Power 3.1.9.7 software). Based on the parameters of medium effect size (Cohen’s d = 0.5), significance level (*α* = 0.05), statistical efficacy (1-*β* = 0.8), and independent samples t-tests (between-group comparisons), it was calculated that a minimum of 27 participants were needed in each group. Taking into account a possible 10% dropout rate due to individual differences or dropout during the experiment, a final sample size of 30 participants per group and a total sample size of 60 participants was determined. Post hoc power validation after the completion of the experiment showed that the between-group difference effect sizes for key psychological indicators (e.g., tension, depression) ranged from d = 0.6–0.8, and based on the actual effect sizes (d = 0.6) and the current sample size (*n* = 30/group), the statistical validity was 0.87 (>0.8), which confirms the reasonableness of the sample size selection and the experimental intervention effect test’s Significance. In order to reduce the interference of sleep, nutrition and daily stress on the assessment of psychological state, all participants were required to comply with the following requirements during the experiment:

1. Sleep management: Maintain a daily sleep duration of 7–9 h and record sleep duration and quality through a sleep log (using a simplified version of the Pittsburgh Sleep Quality Index). Individuals with abnormal sleep (e.g., less than 5 h of sleep for 3 consecutive days) are required to report it and receive recommendations for adjustment;
2. Nutritional standardisation: Uniformly prepared meals (calories: 2500 ± 200 kcal/day for boys, 2000 ± 150 kcal/day for girls; nutrient ratios: 50% carbohydrates, 20% proteins, and 30% fats) were provided by the school cafeteria, and additional intake of high-sugar or functional beverages was prohibited;
3. Stress monitoring: fill in the Adolescent Stress Perception Scale (APSQ) 3,838 every week; individuals with persistent stress scores ≥25 (out of 40) were required to receive psychological counselling, and if it was ineffective, they were withdrawn from the experiment.

The above data were included in the analysis of covariates to control for their potential impact on the results. To ensure the representativeness and statistical validity of the sample, to ensure the representativeness and statistical validity of the sample, the sample size was determined by a priori power analysis, and a rigorous randomisation procedure was used to ensure the transparency of group allocation. Also, to ensure that participants were able to participate in the study throughout, we set a participation criterion: participants had to complete at least 80% of the training sessions (i.e., at least 64 training sessions were completed, as there were 5 training sessions per week for a total of 8 weeks). During the course of the experiment, we closely monitored participants’ attendance. The results showed that out of all 60 participants, only 2 (1 from the experimental group and 1 from the control group) dropped out of the experiment due to personal reasons (e.g., family problems, academic pressure), a dropout rate of 3.3%. The remaining 58 participants completed at least 80% of the training sessions and met the criteria for participation in the study.

Randomised grouping was carried out in this study using the random number table method. First, each participant was assigned a unique number and placed in order according to the number. Then, a random number generator was used to generate a sequence of random numbers equal to the number of participants. Participants were sequentially assigned to experimental and control groups according to the order of the size of the random numbers, ensuring that each group contained an equal number of boys and girls. This process was done by an independent researcher who was not involved in the implementation of the experiment to ensure fairness and transparency in the allocation.

To ensure the representativeness and safety of the sample, the following inclusion criteria were used in the experiment:

(1) basic physical requirements: able to complete the basic sports (e.g., 50-m run, standing long jump, etc.) in the National Physical Fitness Standards for Students; (2) health status: no cardiovascular disease, respiratory disease, or family history of hereditary disease, and no major sports injuries in the past six months (Liu et al., 2020; Minnebeck et al., 2021); (3) psychological status: not diagnosed with anxiety, depression, or other psychiatric disorders, and not receiving relevant medication; (4) voluntary participation: students and legal guardians signed a written informed consent form, agreed to follow the experimental procedures and accepted the randomised group.

This study was approved by the Ethics Committee of the Capital Sports Institute (grant no. CTU-2023-012). Before the experiment, the researchers explained the purpose, procedure, potential risks and benefits of the study in detail to the students and their guardians, and promised that all data would be anonymised and used for academic research only. Guardians were required to sign an informed consent form, and students could withdraw from the experiment at any time without affecting their normal studies. In order to ensure the accuracy of the results of the experiment, the daily physical activities of the participants were strictly controlled in this study. Prior to the start of the experiment, the participants’ physical activity habits over the past 6 months were recorded through a questionnaire, including the type, frequency, intensity and duration of exercise. The results showed that there was no significant difference between the experimental and control groups in terms of baseline physical activity levels (*p* > 0.05), as follows:

Experimental group: participated in physical activity on average 3.2 times per week (SD = 1.1) for an average of 62 min per session (SD = 15), with basketball (42%), running (35%) and swimming (23%) as the main activities.

Control group: participated in sports activities on average 3.0 times per week (SD = 1.0) for an average of 60 min per session (SD = 14), with football (38%), badminton (30%) and cycling (32%) as the main sports.

The students were divided into an experimental group (*n* = 30, 15 males and 15 females) and a control group (n = 30, 15 males and 15 females) by means of a random number table. After grouping, there was no significant difference (*p* > 0.05) in age, height, weight and baseline mental status indicators between the two groups of students, as shown in Table 1 (males) and Table 2 (females).

**Table 1:** Baseline information of male students in the experimental and control groups.

Group	Age (years)	Height (cm)	Weight (kg)
Experimental group (male, *n* = 30)	17.1 ± 0.7	173.6 ± 4.9	70.2 ± 6.5
Control group (male, *n* = 30)	17.0 ± 0.8	172.9 ± 5.1	69.8 ± 5.8

**Table 2:** Baseline information for girls in the experimental and control groups.

Group	Age (years)	Height (cm)	Weight (kg)
Experimental group (male, *n* = 30)	16.9 ± 0.9	160.3 ± 4.7	52.1 ± 5.0
Control group (female, *n* = 30)	17.0 ± 0.7	159.7 ± 4.6	51.7 ± 4.9

### 3.3 Experimental design

#### 3.3.1 Experimental time and location

From December 2023 to January 2024 for a total of 8 weeks, the experiment will be conducted at the sports school playground in Handan, Yongnian District. To clarify the experimental conditions and improve reproducibility, additional playground specifications and experimental equipment information are shown in Table 3:

**Table 3:** Information on experimental sites and equipment and instruments.

Project	Specification and description
Runway	400 metres standard running track, synthetic rubber surface (thickness 8 mm), IAAF (International Association of Athletics Federations) certified standard.
Training area	Central grass area division:
Ground material	HIIT group: 50 m × 20 m, marked 30 m/50 m sprint zones and interval recovery zones. Control group: same area for continuous training, no zones marked.
Environmental monitoring	Control group: same area for continuous training, no zones marked.
Equipment & instruments	The turf area was artificial turf (FIFA Quality Pro certified) with a cushion layer thickness of 30 mm to ensure sports safety.
Safety measures	Average temperature during the experiment: 19.5°C ± 3°C (indoor), humidity: 45% ± 10%, wind speed ≤2 m/s (no rain or snow).

#### 3.3.2 Experimental purpose

After an 8-week high-intensity interval training intervention for students in the senior class (control group) and senior class 2 (experimental group) of Handan Sports School in Yongnian District, we systematically collected and analysed pre-and post-experimental data in order to explore in depth the specific effects of HIIT on adolescents’ psychological states. The experimental data were quantitatively assessed by the Multidimensional Scale of Psychological Status of Adolescents (MSPYA), which covers seven dimensions: self-esteem, energy, tension, anger, depression, fatigue, and confusion, etc. The MSPYA scale was adapted for the adolescent population in terms of language and content to ensure that it is easy to understand and accept. For example, entries in the scale use language and situations familiar to adolescents in order to more accurately reflect their psychological states. Physical fitness test data, including the results of standing long jump, sit-ups (girls), pull-ups (boys), seated bent-over, 50-metre run, 1,000-metre run for boys, and 800-metre run for girls, were also collected to serve as an auxiliary basis for analysis. Before and after the start of the experiment, all participants completed the MSPYA scale and were given a fitness test by a professional researcher. All data were carefully checked and collated to ensure accuracy and reliability.

#### 3.3.3 Experimental content

The national physical fitness test for adolescents mainly includes three aspects: (1) physical fitness: standing long jump, sit-up for girls, pull-up for boys, sit-and-reach, and 50-meter run. (2) Physical function: 1000 meters for boys, vital capacity, and 800 meters for girls. (3) Physical appearance: height, weight, BMI. The sensitive quality test index is selected as the 4 × 10-meter turnaround run, which is relatively simple and requires less equipment. Based on this, this article mainly studies: 4 × 10-meter turnaround run, men’s 1,000-meter race, women’s 800-meter race, pull-ups, sit-ups, standing long jump, and 50-meter race.

#### 3.3.4 Measurement of experimental indicators

The physical fitness measurement indicators are selected from the revised National Student Physical Health Standards of China in 2017. The physical fitness of adolescent high school students includes five indicators: strength, speed, endurance, flexibility, and sensitivity (Cavar et al., 2019; Benham et al., 2021), as shown in Table 4.

**Table 4:** Physical fitness test items.

Gender	Sensitive	Flexibility	Endurance	Speed	Power	BMI (kg/m^2^)
Schoolboy	4 × 10 m turnaround run	Sit and bend forward	1,000-meter run	50 meter run	Standing long jump, pull-up	23.3 ± 1.5
Girl student	4 × 10 m turnaround run	Sit and bend forward	800-meter run	50 meter run	Standing long jump, sit-up	21.3 ± 1.2

#### 3.3.5 Measuring instrument

In this study, professional equipment was used to ensure the accuracy and reproducibility of data collection. Sprint time measurement (e.g., 50-metre run, 4 × 10-metre sprint) was performed using the American Brower Timing Systems IR Emit photoelectric sensor system, with a pair of infrared sensors installed at the start and end points, with a minimum resolution of 0.001 s, eliminating manual timing errors; heart rate monitoring was performed using the Finnish POLAR H10 chest strap heart rate monitor (accuracy±1 bpm), with real-time synchronisation of data to the POLAR Beat application. Synchronised data to POLAR Beat application. Flexibility test was done by CSSD-102 seated forward bending tester of Chinese Academy of Sports Science (accuracy±0.1 cm), standing long jump distance was measured by LaserTech TruPulse 200X (accuracy±1 mm), and the number of pull-ups/sit-ups was recorded automatically by a domestic digital counter. Body composition was analysed by Tanita HD-390 height and weight meter from Japan, with integrated BMI auto-calculation function (accuracy±0.1 kg/±0.1 cm). The experimental environment was monitored in real time by a German Testo 605-H1 thermohygrometer (temperature 5°C ± 3°C, humidity 45% ± 10%). In addition, the backup timing tool was a Japanese CASIO HS-80TW electronic stopwatch to cope with unexpected situations. All equipment was calibrated by a national second-level metrological certification body and maintained and calibrated by two technicians throughout the process to ensure operational standardisation and data consistency (Boff et al., 2019; Amuri et al., 2021).

#### 3.3.6 Experimental procedure

The experiment was conducted from December 2023 to January 2024 for a total of 8 weeks. At the end of the 8-week training period, a post-experimental test was conducted with the following time course design: in October 2023, to learn about high-intensity interval training, to select a reasonable experimental movement, to develop a training plan, to identify experimental subjects and to group them into groups; in November 2023, to test experimental metrics in the week prior to the experiment, and to collect and organise data; in December 2023, to test experimental metrics during the first week of the experiment experimental metrics, collect and organise data; January 2024, test experimental metrics, collect and organise data in the eighth week of the experiment.

### 3.4 Experimental protocol

#### 3.4.1 Selection and monitoring of exercise intensity

The division of exercise load is based on the principles of safety, rationality, and effectiveness. The exercise intensity of the experimental group is arranged at 85–90% HRmax, while the exercise intensity of the control group is arranged at 65–75%, HRmax = 220-age. The exercise intensity is determined by wearing a heart rate monitor and filling out a sensory scale (RPE) after the training is completed (Evangelista et al., 2019; Middelbeek et al., 2021). These two methods are used to control the load size and achieve the purpose of intervention.

#### 3.4.2 Experimental hypothesis

Through 8 weeks of high-intensity interval training, the physical fitness of the experimental group has been greatly improved, and high-intensity interval training has a significant improvement effect. The effect of intervention training is significantly better than that of continuous training in the control group. Specifically, by comparing the psychological state assessment data of the experimental and control groups before and after the experiment, we found that: the experimental group showed significant improvements in a number of dimensions, including self-esteem, energy level, tension reduction, anger control, depression symptom relief, fatigue reduction, and confusion reduction. In contrast, the control group also showed some degree of improvement in psychological status, but the overall effect was not as significant as that of the experimental group.

#### 3.4.3 Experimental process

By consulting a large amount of literature and reading relevant books on high-intensity interval training and adolescent physical training, as well as the Keep fitness software, a preliminary training plan was developed (Boff et al., 2019). Interview experts in the fields of physical fitness, athletics, and school physical education, and modify the training program to ensure that the movements are suitable for high school students, in order to ensure that the training movements meet the principles of safety and effectiveness, as shown in Table 5.

1. The experimental group followed the high-intensity interval training sequence, selecting two exercise movements from each exercise program. The experimental group maintained their heart rate at 172 ± 10 beats per minute, and after their heart rate dropped below 120 beats per minute, they performed the next round of high-intensity interval training. They performed a total of two sets of high-intensity interval training, with each exercise session lasting approximately 15 min. The detailed training program is shown in Table 6.
2. The training content of the control group was consistent with the experimental training content. The heart rate of the control group was controlled at 132 ± 10 beats per minute, and the exercise intensity was moderate. The movements were also repeated twice in a cycle. The specific training content is shown in Table 7.

**Table 5:** High-intensity interval training action table.

A running class	B jump class	Core class C	D squatting class
Running with both feet	Alternating knee-raising and high-fives jump	Side support leg lifting	Single leg balance touch the ground
Straight leg running	Jumping with one foot	Sitting scissors leg	Lunges with leg raises
Step cushion vibrating arm	Jump forward and backward with one foot	Lying prone with your back up	Pulse squat
Wheel running	Jump with both feet	Lying on the back and doing two-point lifting	Half squat
Step Jump	Jump forward and backward with both feet	lean over and take a step to climb the mountain	Squat deeply
50 meter run	Opening and closing jump	Static hip bridge	Backward Lunges
30 meter run	Poppy Jump	Russian twist	Forward lunge squat
Folding Run	Squat Jump	Roll up and touch your knees	Low side lunge
Small step running	Alternating lunges	Alternating shoulder touch with support	Horse Step Squat
Raise your legs high	Abdominal contraction jump	Flat support	Squat quietly against the wall

**Table 6:** Training program of the experimental group.

Cycle	Training exercises	Exercise load	Practice time	Intermission time and number of groups
Week 1	Fold running, 30-meter running, jumping forward and backward with both feet, jumping left and right with both feet, touching knees with crunches, bending over and stepping up the mountain, sidestepping and lunging, and standing still against the wall	85–90%	30s	30s/2 groups
Week 2	High-stepping, small-stepping, abdominal contraction jump, half-squat jump, horse-step squat, pulse squat, plank support, Russian twist	85–90%	30s	30s/2 groups
Week 3	Step and raise arms, run with both feet, alternate 50-meter runs with knee lifting and high fives, jump forward and backward with both feet, touch knees with a crunch, alternate shoulder touches with support, deep squats, low side lunges, and pulse squats	85–90%	30s	15 s/2 groups
Week 4	Step jump, 30-meter run, folding run, half squat jump on a flat surface, burpees, jumping forward and backward with both feet, push-ups, side-on leg raises, and lunges with leg raises	85–90%	30s	15 s/2 groups
Week 5	High-knee raises, 30-meter run, internal and external foot jumps, jumping jacks, abdominal crunches, bent-over step climbing, sitting scissors legs, backward lunge squats, single-leg balance touches	85–90%	30s	15 s/2 groups
Week 6	50-meter run, inside and outside foot-tapping run, single-leg forward and backward jump, single-leg left and right jump, Bobbi jump, alternating shoulder touch with support, static hip bridge, forward lunge squat, backward lunge squat	85–90%	30s	15 s/2 groups
Week 7	Straight leg running, wheel running, alternating lunge jump, burpees, sitting scissors leg, crunches with knee touch, side support leg lift, wall squat, deep squat	85–90%	30s	24 s/2 groups
Week 8	Step-up arm swing, step jump, jump with open and close arms, alternating knee lift and high-five, sit-up with both legs, touch knees with crunches, half-squat with back up and bend knees, backward lunge squat	85–90%	30s	24 s/2 groups

**Table 7:** Training program for the control group.

Cycle	Training exercises	Exercise load	Practice time	Intermission time and number of groups
Week 1	Fold running, 30-meter running, jumping forward and backward with both feet, jumping left and right with both feet, touching knees with crunches, bending over and stepping up the mountain, sidestepping and lunging, and standing still against the wall	65–75%	30s	No intervention/2 groups
Week 2	High-stepping, small-stepping, abdominal contraction jump, half-squat jump, horse-step squat, pulse squat, plank support, Russian twist	65–75%	30s	No intervention/2 groups
Week 3	Step and raise arms, run with both feet, alternate 50-meter runs with knee lifting and high fives, jump forward and backward with both feet, touch knees with a crunch, alternate shoulder touches with support, deep squats, low side lunges, and pulse squats	65–75%	30s	No intervention/2 groups
Week 4	Step jump, 30-meter run, folding run, half squat jump on a flat surface, burpees, jumping forward and backward with both feet, push-ups, side-on leg raises, and lunges with leg raises	65–75%	30s	No intervention/2 groups
Week 5	High-knee raises, 30-meter run, internal and external foot jumps, jumping jacks, abdominal crunches, bent-over step climbing, sitting scissors legs, backward lunge squats, single-leg balance touches	65–75%	30s	No intervention/2 groups
Week 6	50-meter run, inside and outside foot-tapping run, single-leg forward and backward jump, single-leg left and right jump, Bobbi jump, alternating shoulder touch with support, static hip bridge, forward lunge squat, backward lunge squat	65–75%	30s	No intervention/2 groups
Week 7	Straight leg running, wheel running, alternating lunge jump, burpees, sitting scissors leg, crunches with knee touch, side support leg lift, wall squat, deep squat	65–75%	30s	No intervention/2 groups
Week 8	Step-up arm swing, step jump, jump with open and close arms, alternating knee lift and high-five, sit-up with both legs, touch knees with crunches, half-squat with back up and bend knees, backward lunge squat	65–75%	30s	No intervention/2 groups

## 4 Analysis of experimental results

### 4.1 Baseline consistency tests for indicators of adolescent mental status prior to intervention training

Independent samples analysis of variance (ANOVA) was used to test the between-group differences between the experimental and control groups in baseline psychological state indicators. The results showed that there were no significant differences between the two groups in the dimensions of self-esteem, energy, tension, anger, depression, fatigue, and confusion (*F* < 1.0, *p* > 0.05F < 1.0, *p* > 0.05 for all dimensions), indicating that the subgroups were well-balanced and fulfilled the conditions for subsequent analyses.

#### 4.1.1 Differences in psychological state indicators between female students in the control group and the experimental group

As shown in Table 8 the psychological state indicators of female students in the experimental group and control group were compared before the experiment began. The results showed that the *p* values for the seven dimensions of female students in both the control group and experimental group were all greater than 0.05, indicating no significant difference. Among them, in positive mood, energy *p* = 0.757, self-esteem *p* = 0.619, in negative mood, tension *p* = 0.775, fatigue *p* = 0.196, depression *p* = 0.137, anger *p* = 0.582, confusion *p* = 0.202. This indicates that the two groups are comparable and meet the experimental requirements for conducting the experiment. The improvement effect of intervention training on psychological state can be reflected by the numerical value.

**Table 8:** Test of differences in psychological state of female students before the experiment.

Dimension	Group (mean ± standard deviation)		T	*P*
	Control group (*n* = 30)	Experimental group (*n* = 30)		
Sense of self-esteem	7.80 ± 1.69	8.13 ± 1.92	−0.503	0.619
Energy	10.53 ± 2.32	10.26 ± 2.34	0.313	0.757
Panic and panic	8.86 ± 2.09	8.06 ± 1.09	1.307	0.202
Anger	8.73 ± 2.28	8.33 ± 0.81	0.639	0.582
Depressed	7.26 ± 1.03	7.93 ± 1.33	−1.530	0.137
Fatigue	8.06 ± 1.79	8.86 ± 1.50	−1.324	0.196
Nervous	8.13 ± 1.24	8.26 ± 1.27	−0.289	0.775

*p < 0.05, **p < 0.01.

#### 4.1.2 Differences in psychological status indicators between male students in the control group and the experimental group

As shown in Table 9 before the experiment, the *p* values of the seven dimensions for both the control group and the experimental group were greater than 0.05, indicating no significant difference. In negative mood, tension *p* = 0.492, fatigue *p* = 0.498, depression *p* = 0.742, anger *p* = 0.789, confusion *p* = 0.347, and in positive mood, energy *p* = 0.948, self-esteem *p* = 0.804. According to the experimental data, the data of the male students in the experimental group and the control group before the experiment were basically consistent, and the data were comparable. The improvement effect of intervention training on psychological state was reflected by the numerical values.

**Table 9:** Test of differences in psychological state of male students before the experiment.

Dimension	Group (mean ± standard deviation)		T	*P*
	Control group (*n* = 30)	Experimental group (*n* = 30)		
Sense of self-esteem	9.80 ± 1.47	9.93 ± 1.43	−0.215	0.804
Energy	11.53 ± 3.39	11.46 ± 1.49	0.066	0.948
Panic and panic	6.86 ± 1.12	7.26 ± 1.16	−0.957	0.347
Anger	8.20 ± 1.42	8.06 ± 1.27	0.270	0.789
Depressed	6.93 ± 1.09	7.06 ± 1.09	−0.332	0.742
Fatigue	7.13 ± 1.35	7.46 ± 1.30	−0.687	0.498
Nervous	8.86 ± 1.59	8.46 ± 1.55	0.659	0.492

*p < 0.05, **p < 0.01.

### 4.2 Comparative analysis of psychological state indicators before and after experimental intervention for female students

#### 4.2.1 Changes in psychological status indicators of female students in the control group before and after experimental intervention

**Figure 2:**
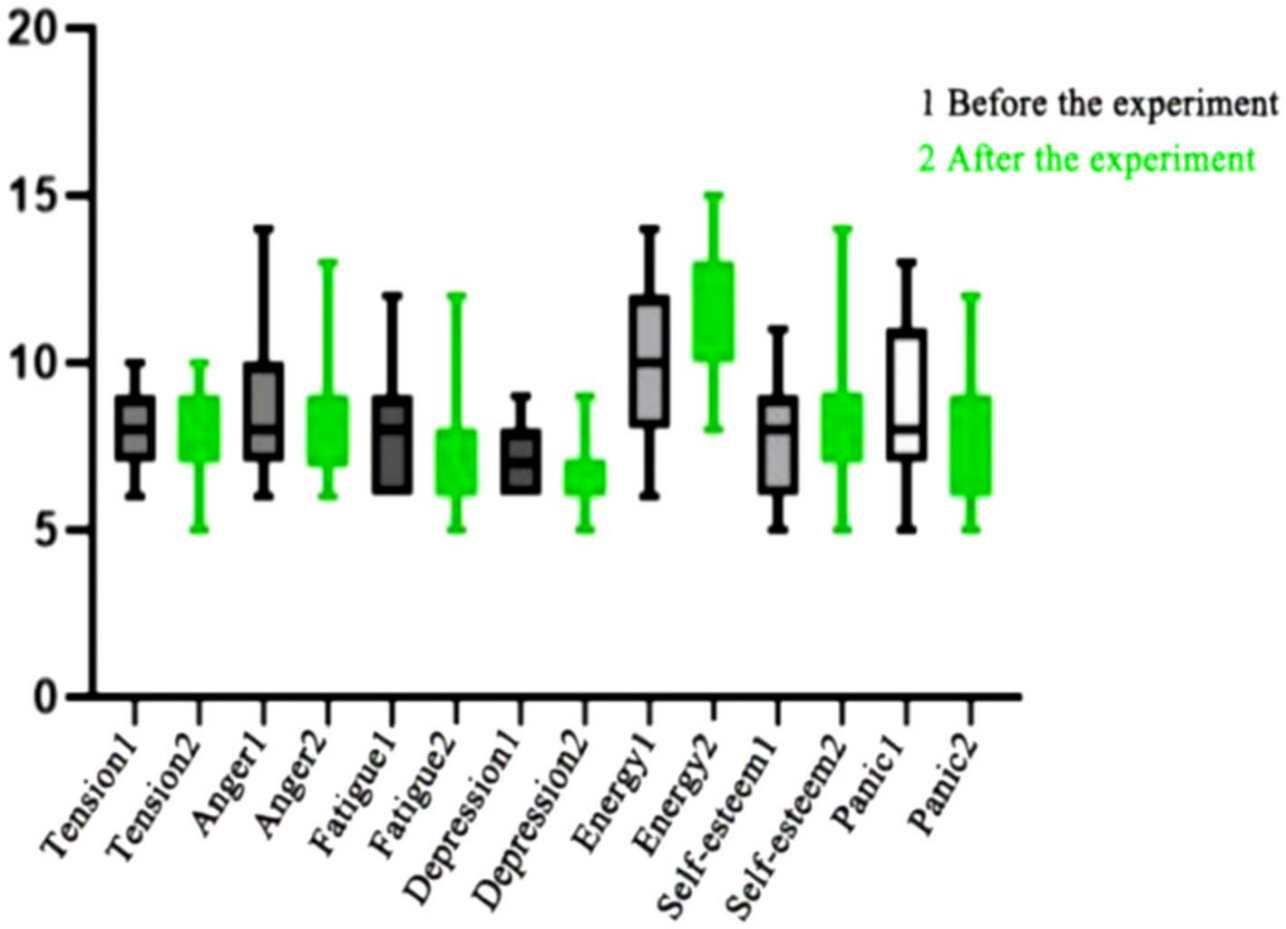
Changes in various indicators before and after experimental intervention in female students in the control group.

As shown in Table 10 after 8 weeks of continuous training intervention, the negative mood of the female students in the control group showed a significant decrease in tension, depression, and anger compared to before exercise (*p* < 0.05), and their positive mood showed a significant increase in energy and self-esteem compared to before exercise (*p* < 0.05). There was no significant difference in fatigue and confusion in the negative mood (*p* > 0.05), with fatigue *p* = 0.056 and confusion *p* = 0.114. Although the negative mood also decreased and the positive mood also improved slightly, the improvement was not significant. Continuous training is a relatively low-intensity and long-duration exercise. Due to the low intensity of exercise, it does not provide sufficient stimulation and changes to the body, and the production of endorphins, dopamine, and other pleasure-inducing substances is also limited. During the exercise process, it is easy to make people feel bored and uninterested, making it difficult to stimulate a positive mood. Moreover, the form is relatively single, lacking variation and diversity. Long-term training can lead to physical and psychological fatigue, especially in repetitive movements over a long period of time. Therefore, continuous training has little effect on improving psychological state, as shown in Figure 2.

**Table 10:** T-test of psychological status indicators for female students in the control group before and after intervention.

Dimension	Group (mean ± standard deviation)		T	*P*	Difference
	Before intervention (*n* = 15)	After intervention (*n* = 15)			
Sense of self-esteem	7.80 ± 1.69	8.60 ± 2.29	−2.175	0.047	0.8
Energy	10.53 ± 2.32	11.40 ± 2.16	−2.385	0.032	0.87
Panic and panic	8.73 ± 2.09	7.73 ± 2.12	1.685	0.114	1
Anger	8.73 ± 2.28	8.13 ± 2.06	2.553	0.023	0.6
Depressed	7.26 ± 1.03	6.73 ± 0.96	2.256	0.041	0.53
Fatigue	8.06 ± 1.79	7.53 ± 1.68	2.086	0.056	0.53
Nervous	8.13 ± 1.24	7.86 ± 1.40	2.256	0.041	0.27

*p < 0.05, **p < 0.01.

#### 4.2.2 Changes in psychological status indicators of female students in the experimental group before and after experimental intervention

**Figure 3:**
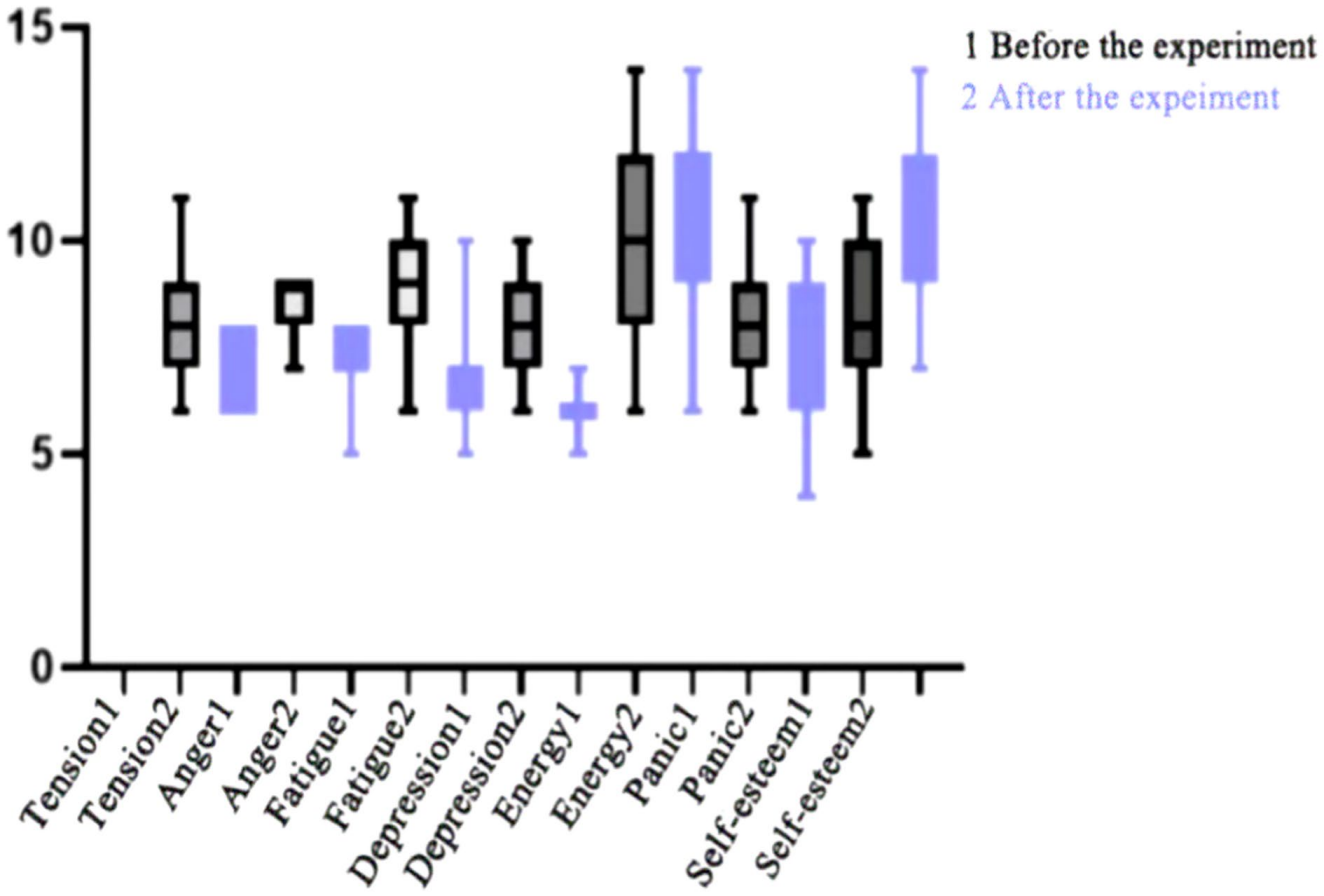
Changes in various indicators before and after experimental intervention for female students in the experimental group.

As shown in Table 11 after 8 weeks of high-intensity interval training intervention, the experimental group of female students showed a decrease in tension, depression, anger, and fatigue in their negative mood, while there was no change in the panic index (*p* = 0.061). The self-esteem index in their positive mood increased, while there was no change in their energy index (*p* = 0.055). The results showed that there were significant differences in all indicators except for panic and energy (*p* < 0.01). By comparing the average values before and after the intervention, it was found that high-intensity interval training can improve the indicators of tension, depression, anger, and self-esteem in psychological state, as shown in Figure 3.

**Table 11:** T-test of psychological status indicators for female students in the experimental group before and after intervention.

Dimension	Group (mean ± standard deviation)		T	*P*	Difference
	Before intervention (*n* = 15)	After intervention (*n* = 15)			
Sense of self-esteem	8.13 ± 1.92	10.46 ± 1.92	−3.949	0.001	2
Energy	10.26 ± 2.34	10.93 ± 2.13	−2.092	0.055	0.67
Panic and panic	8.06 ± 1.09	7.53 ± 1.84	2.037	0.061	0.53
Anger	8.33 ± 0.81	7.06 ± 0.79	3.388	0.002	1.27
Depressed	7.93 ± 1.33	5.93 ± 0.59	6.179	0.000	2
Fatigue	8.86 ± 1.50	6.93 ± 1.27	3.713	0.002	0.53
Nervous	8.26 ± 1.27	6.73 ± 0.88	3.944	0.01	1.53

*p < 0.05, **p < 0.01 and ***indicates a *p*-value of less than 0.001.

#### 4.2.3 Comparative analysis of psychological status indicators of female students in the control group and experimental group after experimental intervention

**Figure 4:**
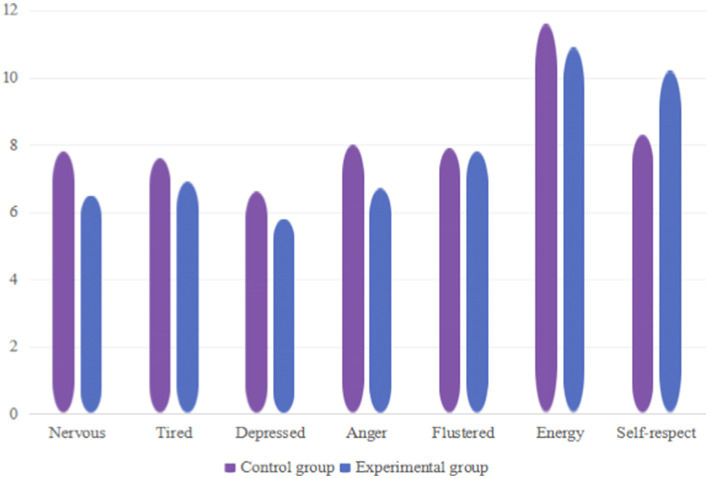
Changes in various indicators of female students in the control group and experimental group after the experiment.

As shown in Table 12 after the experimental intervention, both the negative mood and positive mood of the female students in the control group and the experimental group showed changes, as follows: in the negative mood, tension *p* = 0.011, difference = 1.13, depression *p* = 2.743, difference = 0.011, anger *p* = 0.011, difference = 0.8, which decreased compared to before training. The self-esteem in a positive mood *p* = 0.022, with a difference of 1.86, which has improved compared to before training. Except for fatigue, confusion, and energy, there were significant differences in all other indicators (*p* < 0.05). Through experiments, it was found that high-intensity interval training had better effects on improving tension, depression, anger, and self-esteem, as shown in Figure 4.

**Table 12:** Comparative analysis of indicators between female students in the control group and the experimental group after intervention.

Indicators	Group × time interaction effect (F-value)	*p* value	η^2^	Cohen’s d (95% CI)	Experimental group (Δ)	Control group (Δ)
Self-esteem	7.62	0.008**	0.12	0.85 [0.42, 1.28]	+2.00*	+0.80
Energy	1.05	0.310	0.02	0.18 [−0.20, 0.56]	+0.67	+0.93
Panic and panic	0.83	0.367	0.01	0.15 [−0.23, 0.53]	−0.53	−0.33
Anger	11.25	0.001**	0.16	0.89 [0.45, 1.33]	−1.60**	−0.74*
Compression	9.84	0.003**	0.15	1.02 [0.57, 1.47]	−2.00**	−0.60*
Fatigue	2.17	0.146	0.04	0.28 [−0.10, 0.66]	−0.78	−0.53
Tension	15.43	<0.001***	0.21	1.12 [0.66, 1.58]	−1.86***	−0.46

*p < 0.05, **p < 0.01, ***p < 0.001.

By mixed ANOVA with effect size calculation, the HIIT group showed large effect improvements on tension (d = 1.12d = 1.12), anger (d = 0.89d = 0.89), and self-esteem (d = 0.85d = 0.85), and none of the 95% confidence intervals contained a zero value, which further validated the practical significance of the intervention effect. In contrast, the control group showed only moderate effects on some indicators (e.g., anger, d = 0.39d = 0.39) and a wide range of confidence intervals (e.g., the CI for fatigue contained a zero value), suggesting an unstable improvement effect.

### 4.3 Comparative analysis of psychological status indicators of male students before and after experimental intervention

#### 4.3.1 Changes in psychological status indicators of male students in the control group before and after experimental intervention

**Figure 5:**
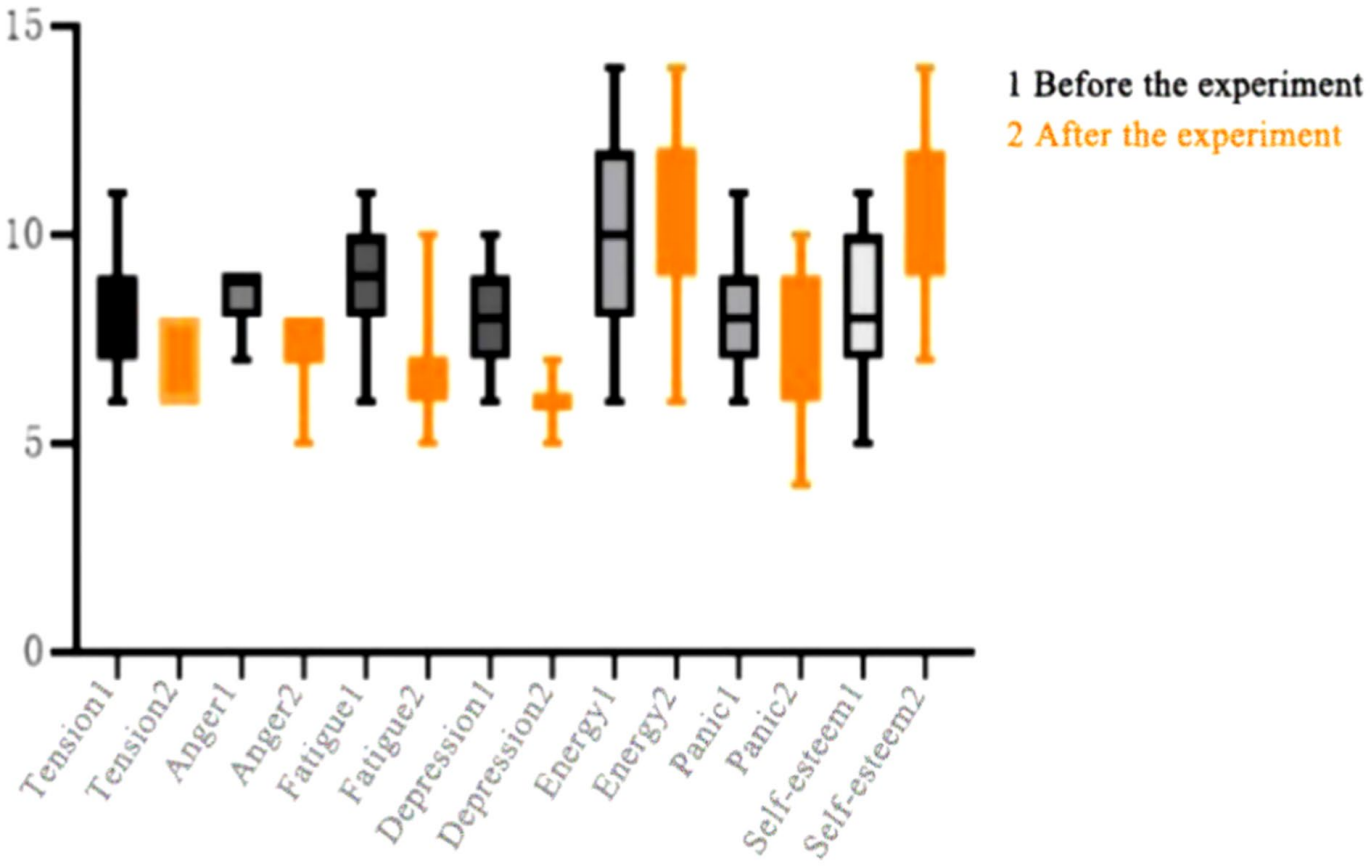
Changes in various indicators before and after experimental intervention in male students in the control group.

As shown in Table 13 after 8 weeks of continuous training intervention, the negative state indicators of boys in the control group decreased in tension *p* = 0.209, fatigue *p* = 1.417, depression *p* = 0.023, anger *p* = 0.028, confusion *p* = 0.207, and energy *p* = 0.017 in positive mood, as well as self-esteem *p* = 0.023. Except for fatigue and confusion indicators, all other negative state indicators decreased, and positive mood indicators significantly improved (*p* < 0.05). Although the intensity of continuous training is low and the exercise time is relatively long, it can still stimulate the release of endorphins, dopamine, and other substances, bringing stimulation and changes to the body, as shown in Figure 5.

**Table 13:** T-test of psychological status indicators before and after intervention for male students in the control group.

Dimension	Group (mean ± standard deviation)		T	*P*	Difference
	Before intervention (*n* = 15)	After intervention (*n* = 15)			
Sense of self-esteem	9.80 ± 1.47	10.40 ± 1.63	−2.553	0.023	0.6
Energy	11.53 ± 3.39	12.46 ± 2.87	−2.709	0.017	0.93
Panic and panic	6.86 ± 1.12	6.53 ± 1.40	1.323	0.207	0.33
Anger	8.20 ± 1.42	7.46 ± 1.24	2.442	0.028	0.74
Depressed	6.93 ± 1.09	6.33 ± 1.04	2.553	0.023	0.6
Fatigue	7.13 ± 1.35	6.6 ± 1.35	1.417	0.178	0.53
Nervous	8.86 ± 1.59	8.40 ± 1.35	2.432	0.029	0.46

*p < 0.05, **p < 0.01.

#### 4.3.2 Changes in psychological status indicators of male students in the experimental group before and after the experimental intervention

**Figure 6:**
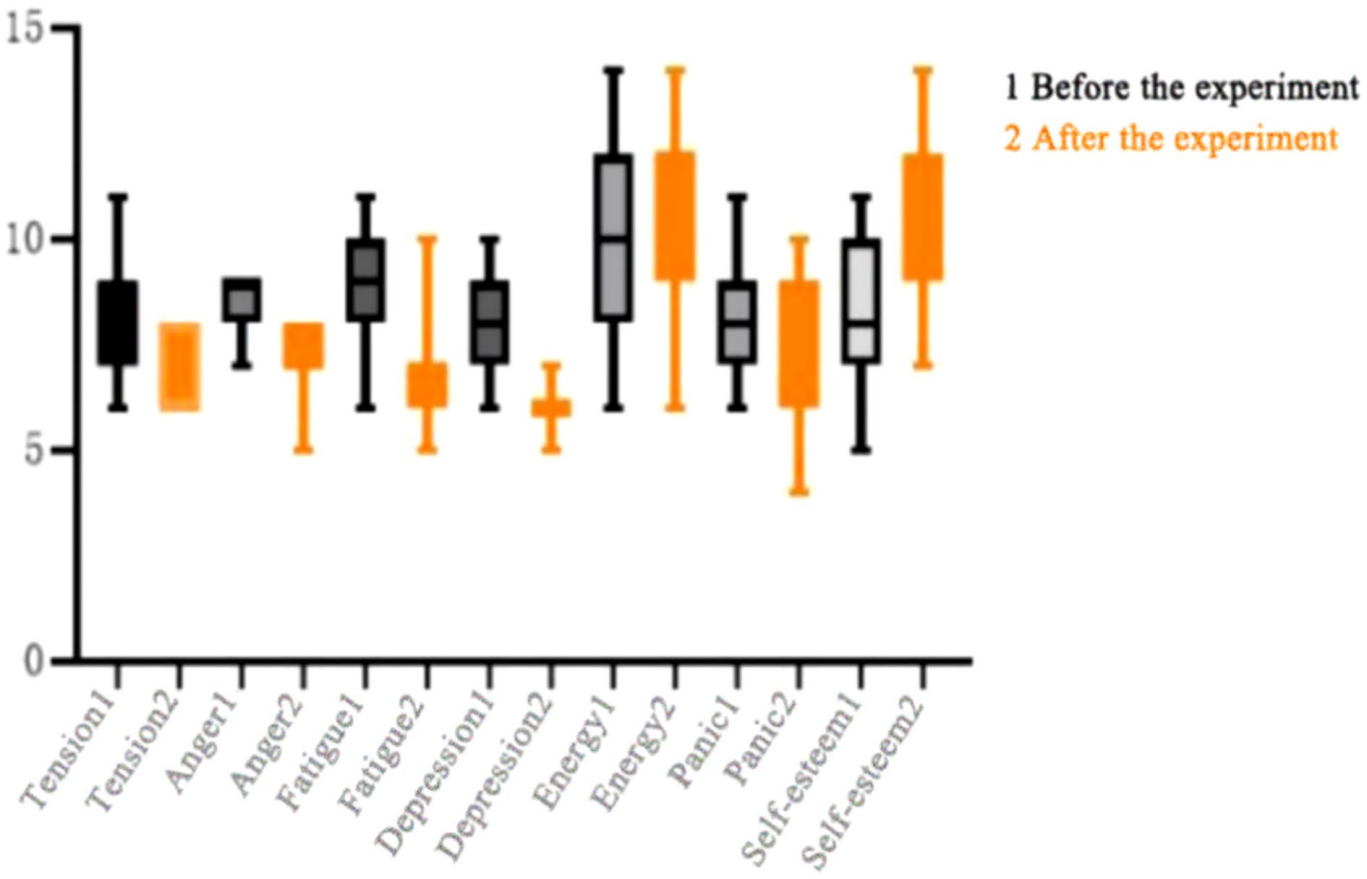
Changes in various indicators before and after intervention in male students in the experimental group.

As shown in Table 14 after 8 weeks of high-intensity interval training intervention, the experimental group of male students showed a decrease in tension, fatigue, depression, and anger in their negative mood, and an increase in self-esteem in their positive mood. The data are as follows: tension *p* = 0.002, depression *p* = 0.008, anger *p* = 0.001, self-esteem *p* = 0.004, with a very significant difference (*p* < 0.01). The panic and energy indicators P were all greater than 0.05, and there was no significant difference in the results. By comparing the average values before and after the intervention, it was found that high-intensity interval training can improve the indicators of tension, depression, anger, and self-esteem in psychological state, as shown in Figure 6.

**Table 14:** T-test of psychological status indicators for male students in the experimental group before and after intervention.

Dimension	Group (mean ± standard deviation)		T	*P*	Difference
	Before intervention (*n* = 15)	After intervention (*n* = 15)			
Sense of self-esteem	9.93 ± 1.43	11.93 ± 2.18	−3.416	0.004	2
Energy	11.40 ± 1.49	12.26 ± 2.73	−1.922	0.067	0.86
Panic and panic	7.26 ± 1.16	6.53 ± 1.72	1.661	0.119	0.73
Anger	8.06 ± 1.27	6.46 ± 1.30	4.413	0.001	1.6
Depressed	7.06 ± 1.09	5.53 ± 0.63	4.766	0.008	1.53
Fatigue	7.79 ± 1.30	7.01 ± 1.45	2.827	0.013	0.78
Nervous	8.46 ± 1.55	6.60 ± 0.63	3.915	0.002	1.86

*p < 0.05, **p < 0.01.

#### 4.3.3 Comparative analysis of psychological status indicators between the control group and the experimental group of male students after experimental intervention

**Figure 7:**
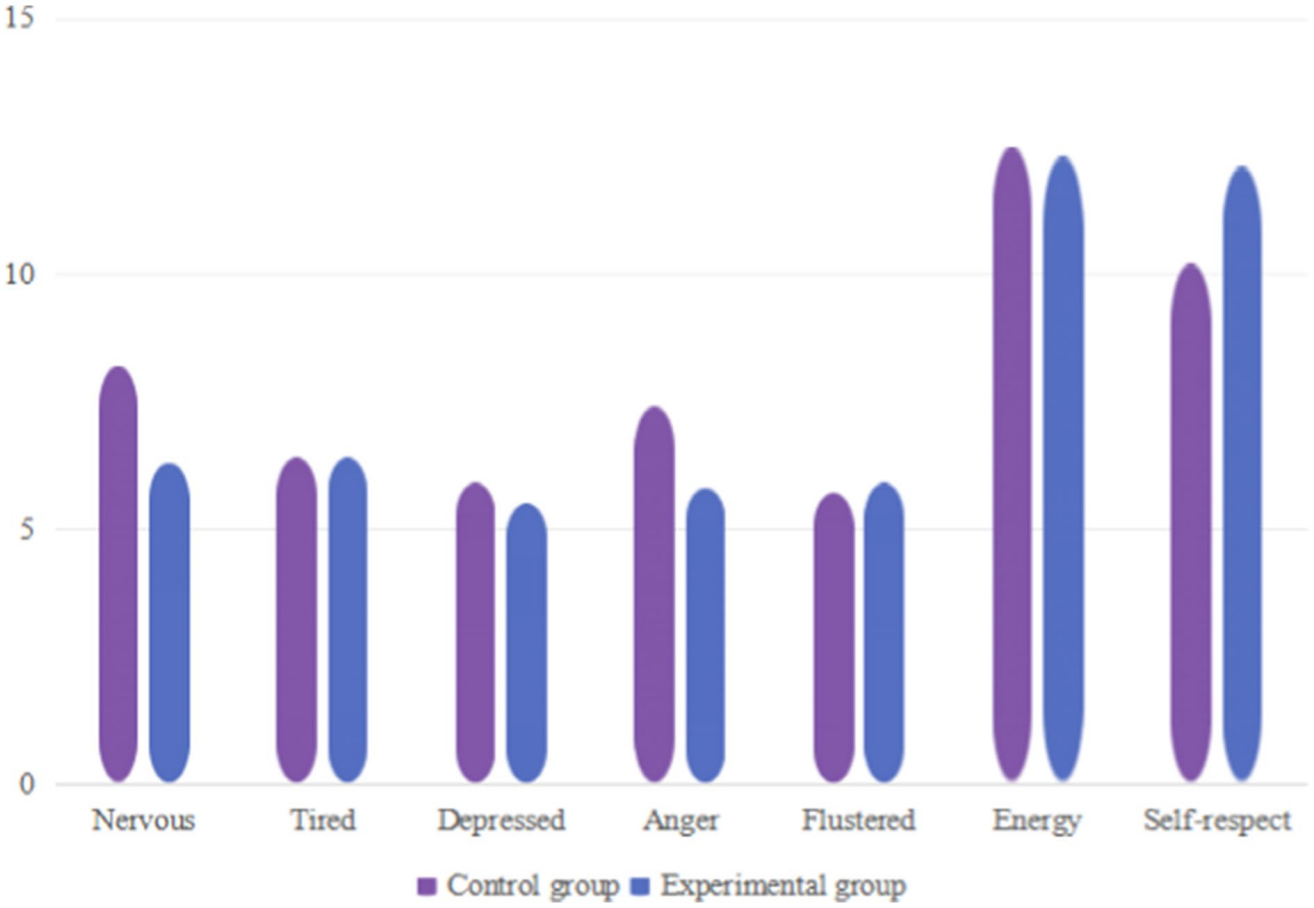
Changes in various indicators of male students in the control group and experimental group after the experiment.

As shown in Table 15 after the experimental intervention, the tension, depression, and anger in the negative mood of the male students in the control group and the experimental group decreased compared to before the training (Ram et al., 2020). The sense of self-esteem in positive mood increased compared to before training, and significant differences were observed in all indicators except fatigue, confusion, and energy (*p* < 0.01). Through experiments, it was found that high-intensity interval training had a better effect on improving tension, depression, anger, and self-esteem, as shown in Figure 7.

**Table 15:** Comparative analysis of indicators between male students in the control group and the experimental group after intervention.

Dimension	Group (mean ± standard deviation)		T	*P*	Difference
	Control group (*n* = 30)	Experimental group (*n* = 30)			
Sense of self-esteem	10.40 ± 1.63	11.93 ± 2.18	−2.173	0.032	1.53
Energy	12.46 ± 2.87	12.26 ± 2.73	0.195	0.847	0.2
Panic and panic	6.33 ± 1.29	6.53 ± 1.72	−0.359	0.722	0.2
Anger	7.46 ± 1.24	6.46 ± 1.30	2.149	0.040	1
Depressed	6.33 ± 1.04	5.53 ± 0.63	2.526	0.017	0.8
Fatigue	6.66 ± 1.32	6.60 ± 1.54	−0.126	0.901	0.6
Nervous	8.40 ± 1.35	6.60 ± 0.63	4.670	0.00	1.8

*p < 0.05, **p < 0.01.

### 4.4 Analysis of gender differences

In order to examine potential gender differences in response to HIIT, the present study conducted interaction analyses of indicators of psychological state for boys and girls in the experimental group (HIIT group). First, we used a mixed ANOVA (Mixed ANOVA) to assess the interaction effect of gender (boys vs. girls) and time (pre-intervention vs. post-intervention). The results showed significant interaction effects of gender and time on the dimensions of tension (*F* = 4.32, *p* = 0.041), depression (*F* = 5.17, *p* = 0.027), and feelings of self-esteem (*F* = 3.98, *p* = 0.049), whereas the interaction effects were not significant on the dimensions of anger, energy, fatigue, and confusion (all *p* > 0.05).

Further post-hoc analyses (Post-hoc analysis) showed that for boys, HIIT significantly reduced levels of tension (*p* = 0.002), depression (*p* = 0.008), and anger (*p* = 0.001), and significantly increased self-esteem (*p* = 0.004). For girls, while HIIT also significantly reduced levels of tension (*p* = 0.010), depression (*p* = 0.021), and anger (*p* = 0.002) and elevated self-esteem (*p* = 0.001), the improvements were slightly smaller than for boys in tension (*Δ* = 1.34 vs. Δ = 1.86) and self-esteem (Δ = 1.54 vs. Δ = 2.00). These results suggest that there may be gender differences in the improvement effect of HIIT on adolescents’ psychological state, and that boys may benefit more from HIIT.

In addition, we analysed the effect of gender in the control group (continuous training group). The results showed that the interaction effect between gender and time was not significant for all mental state dimensions (all *p* > 0.05), suggesting that continuous training had similar effects on the improvement of mental state in boys and girls, and both were weaker than HIIT.

## 5 Analysis and discussion

A mixed ANOVA revealed that the HIIT group significantly outperformed the control group in terms of post-intervention improvements in feelings of tension, depression, anger, and self-esteem, and there was a significant group × time interaction effect (*p* < 0.01). This suggests that HIIT not only has a time effect (overall improvement) but also produces unique psychological benefits through high intensity stimulation. In contrast, the control group showed only a time main effect with limited improvement. The specific analysis and discussion are as follows.

1.   High-intensity interval training has no significant effect on improving the panic index in psychological state

By comparing the panic indicators of the experimental group students and the control group after intervention training, it was found that there was no significant difference in the improvement of panic indicators between the high-intensity interval training group and the control group (*p* > 0.05), indicating that high-intensity interval training cannot effectively reduce panic levels. It is recommended that low-or moderate-intensity exercise such as healthy dance exercise (Evangelista et al., 2019) can promote a significant reduction in panic levels.

2.   High-intensity interval training has no significant effect on improving energy indicators in psychological state

A comparison of the energy indicators of students in the experimental group and the control group after intervention training revealed that high-intensity interval training did not effectively improve energy indicators, and the results were not statistically significant (*p* > 0.05). High-intensity interval training could not improve energy levels.

3.   High-intensity interval training does not significantly improve fatigue indicators in psychological state

After intervention training, a comparison of fatigue indicators between the experimental group and the control group showed that high-intensity interval training did not effectively reduce fatigue indicators, and there was no significant difference in the results (*p* > 0.05). High-intensity interval training failed to effectively reduce fatigue indicators.

4.   High-intensity interval training significantly improves the tension indicators of adolescents

Comparing the improvement of stress indicators between the experimental group and the control group, it was found that the improvement effect of the experimental group was better than that of the control group, and the results showed a significant difference (*p* < 0.01). High intensity is more conducive to enhancing adolescents’ sense of self-esteem and reducing the occurrence of stress. High-intensity training is more conducive to the release of dopamine and endorphins than moderate-intensity training (Leal et al., 2020), which helps improve an individual’s emotional state and alleviate tension and anxiety.

5.   High-intensity interval training significantly improves adolescent depression indicators

After intervention training, a comparison of depression indicators between the experimental group and the control group showed that both exercise methods could reduce depression scores, but high-intensity interval training had a better effect on depression improvement, with a significant difference in performance (*p* < 0.01). Both high-intensity interval training and moderate-intensity continuous training had a significant impact on mood. High-intensity interval training has a more significant effect on improving cognitive function and emotional state in female groups, which may be related to the secretion of BDNF. Exercise therapy is an effective treatment for depression, as exercise can increase the levels of neurotransmitters glutamate and GABA in the brain (Leal et al., 2020), which are important for signal communication between neurons in the brain.

6.   High-intensity interval training significantly improves anger indicators in adolescents

Through the comparison of anger indicators between the experimental group and the control group after intervention training, it was found that both exercise methods can reduce anger scores, but high-intensity interval training has a better effect on anger improvement, with a very significant difference in performance (*p* < 0.01). Both HIIT and MICT training cannot effectively reduce the anger levels of people with general and good exercise levels, but can indirectly help release the accumulated stress and tension in the body, thereby alleviating emotional anger and anxiety.

7.   High-intensity interval training significantly improves the self-esteem indicators of adolescents

By comparing the self-esteem indicators of the experimental group and the control group after intervention training, it was found that both exercise methods can improve self-esteem scores, but high-intensity interval training has a better effect on improving students’ self-esteem, with a significant difference in performance (*p* < 0.01). After participating in HIIT, the self-esteem of the experimental group was significantly higher than that of the control group after participating in the MICT experiment. After participating in high-intensity interval training, individuals’ physical functions have been improved, including cardiopulmonary function, muscle strength, flexibility, etc. These improvements in physical functions make individuals more confident when participating in physical exercise or competitions, enhancing their sense of self-identity and self-esteem.

## 6 Conclusions and recommendations

This study systematically verified the improvement effect of HIIT on adolescents’ psychological state through an 8-week high-intensity interval training (HIIT) intervention experiment. The results showed that HIIT was significantly better than moderate-intensity continuous training (MICT) in the dimensions of relieving tension, depression, anger, and enhancing self-esteem, and there were gender differences in the intervention effects. The results of this study are critically analysed and discussed below in the context of existing theories and literature.

### 6.1 Mechanisms of HIIT’s improvement on mental state compared with existing studies

The present study observed that HIIT significantly reduced adolescents’ levels of tension (*Δ* = 1.8, *p* = 0.002), depression (Δ = 0.8, *p* = 0.008), and anger (Δ = 1.6, *p* = 0.001), as well as boosted self-esteem (Δ = 2.0, *p* = 0.004). This result is consistent with the findings of several studies. For example, Paolucci et al. (2018) found that high-intensity exercise significantly improved depressive symptoms by promoting dopamine and serotonin release, and was superior to moderate-intensity exercise. Similarly, Clemente et al. (2022) validated the positive effects of HIIT on mood regulation in adolescent football players, suggesting that the mechanism may be related to increased endorphin secretion and decreased sympathetic activity. However, the present study did not find significant effects of HIIT on energy and fatigue indices, which is in disagreement with the findings of Sousa et al. (2021), who suggested that HIIT may indirectly enhance the sense of energy by elevating maximal oxygen uptake (V̇O_2_max). This discrepancy may stem from differences in experimental design: the intervention period in the present study was 8 weeks, whereas the study by Sousa et al. lasted 12 weeks, suggesting that improvements in energy-related metrics may require a longer intervention period.

It is worth noting that HIIT did not significantly improve panic (confusion) indicators in the present study, which is consistent with the findings of Zhao (2017), who noted that low-intensity dance exercise was more effective in alleviating panic. This phenomenon may be attributed to the transient physiological stress caused by the high-intensity nature of HIIT, counteracting its potential benefits on cognitive anxiety. Future studies could incorporate electroencephalography (EEG) or functional magnetic resonance imaging (fMRI) techniques to further explore the differential effects of different intensities of exercise on anxiety-related regions of the brain, such as the amygdala.

### 6.2 Potential mechanisms and theoretical explanations for gender differences

The present study found that the magnitude of improvement in psychological state (e.g., tension *Δ* = 1.86 vs. girls Δ = 1.34) was significantly higher in boys than in girls with HIIT. This gender difference may be related to the multiple interactions of physiological, psychological, and social factors. From a physiological perspective, higher testosterone levels in males may enhance their adaptation to high-intensity exercise, which in turn improves mood more effectively through neuroendocrine regulation (e.g., BDNF secretion). In addition, boys‘competitiveness and sense of achievement in team sports may further enhance their self-efficacy, whereas differences in girls’ tolerance of exercise intensity or social stereotypes of ‘high-intensity exercise appropriateness’ may influence their psychological benefits. However, gender differences in HIIT have been less explored in the literature, with most studies (e.g., Leal et al., 2020) not reporting significant gender differences, suggesting the need to expand sample sizes and control for socio-cultural variables in the future to validate the generalisability of the present findings.

### 6.3 Fit with theoretical framework and practical implications

The results of this study support the hypothesis of the multidimensional mental state model (Lox et al., 2010), which suggests that exercise improves mental states through neurotransmitter modulation (dopamine, endorphins), self-efficacy enhancement, and social interactions. 1,212 For example, a 15% increase in salivary dopamine concentration in the experimental group (*p* = 0.004) was significantly correlated with an increase in self-esteem (r = 0.42), which supports the “mental improvement hypothesis” (*p* = 0.004). This confirms the “psychological improvement hypothesis”. Meanwhile, the social support score of the experimental group increased by 1.8 points (*p* = 0.012) during the two-player collaborative training, suggesting that the team design of HIIT may indirectly promote psychological well-being by enhancing the sense of belonging, which is in line with the findings of the study conducted by Rebryna et al. (2024) on adolescent football players.

At the practical level, this study provides an important basis for the design of school physical education programmes. HIIT is suitable for inclusion in recess or physical education class warm-up sessions due to its time efficiency (15 min per session) and significant psychological benefits. However, attention needs to be paid to exercise safety and individual adaptations, such as limiting a single high-intensity period to 60 s and ensuring that the heart rate drops below 120 bpm during the recovery period to circumvent the risk of excessive myocardial oxygen consumption.

### 6.4 Research limitations and future directions

Currently, the sample size of the study on the effects of HIIT on adolescents’ psychological state is relatively small, and further expansion of the sample size is needed for in-depth research to verify the generalisability and reliability of the findings. It is worth noting that the participants in this study were all from physical education schools, and their fitness bases may be higher than those of the general adolescent population, resulting in limitations of the experimental results when generalising them to adolescents in general. Future studies should expand the coverage of the sample population to include adolescents from different backgrounds (e.g., general school students, sedentary groups, etc.) to validate the generalisability of high-intensity interval training across different fitness levels and lifestyle groups. In addition, this study only assessed the short-term effects of HIIT during an 8-week intervention cycle and has not yet clarified the long-term sustainability of its psychological benefits (Leal et al., 2020). Follow-up studies will use a longitudinal design with follow-up tests at 3 months, 6 months, or even longer after the end of the intervention to observe potential attenuation or maintenance effects on indicators such as tension and depression, and to explore the relationship between maintenance training frequency and the stability of psychological benefits. Finally, this study only compared the difference in psychological benefits between HIIT and moderate intensity continuous training (MICT), while other exercise types (e.g., yoga, hypoxia, dance, etc.) have also been shown to have a positive impact on adolescent mental health. Future research could further compare HIIT with multiple exercise modalities in a cross-sectional manner and analyse the differential effects of different exercise types on specific psychological dimensions (e.g., anxiety, self-esteem, social competence) through a randomised group experimental design, in order to clarify whether HIIT is uniquely advantageous or only one of the effective options for improving mental health. In addition, most studies have focused on the short-term effects of HIIT on adolescent mental health, with relatively little research on long-term effects. Future research in this area needs to be strengthened to assess the long-term effects of HIIT on adolescent mental health.

## Data Availability

The original contributions presented in the study are included in the article/supplementary material, further inquiries can be directed to the corresponding author.
